# Effect of cultured white soft cheese on the histopathological changes in the kidneys and liver of albino rats

**DOI:** 10.1038/s41598-022-06522-y

**Published:** 2022-02-15

**Authors:** Khaled H. Salman, Fatma Abo Zakaib Ali, Ruwaida Elhanbaly

**Affiliations:** 1 Department of Dairy Science, Faculty of Agriculture, AlAzhar University, Assiut, 71524 Egypt; 2 Department of Pathology and Clinical Pathology, Faculty of Veterinary Medicine, Sohag University, Sohâg, 82524 Egypt; 3 Department of Anatomy and Histology, Faculty of Veterinary Medicine, Assiut University, Assiut, 71515 Egypt

**Keywords:** Biotechnology, Microbiology, Pathogenesis

## Abstract

Three different types of lactic acid bacteria (*Lactobacillus helveticus*, *Lactobacillus rhamnosus* and *Streptococcus thermophilus* S_3855_) were used to manufacture white soft cheese. The resultant white soft cheeses were pickled for 28 days at refrigerator temperatures and were fed to the experimental rats. The chemical and microbiological analyses of white soft cheese were conducted at different storage periods (fresh, 7 days, 14 days, 21 days, and 28 days). The pH values and protein content of white soft cheese gradually decreased during the storage peroid. Conversely, the moisture content, titratable acidity, and fat/DM % of white soft cheese were found to increase with of the increase in pickling periods of up to 28 days. Microbiologically, the total viable count of bacteria in the control samples was lower than that in the other treatments. Furthermore, the treatments containing the *L. helveticus* and *L. rhamnosus* strains had the highest lactoacilli counts whereas the treatment containing the *S. thermophilus* strain had the highest streptococci counts. Twenty-five male Albino rats were used for experiemntal technique. Rats were fed with 70% basal diet with addition of 30% white soft cheese. Several pathological findings were present in all experimental groups apart from the control rats, and the kidney samples exhibited renal vascular congestion especially in the cortical area. The changes of the glomeruli comprise atrophy, distortion, hypocellularity of the glomerular tuft, and focal lymphoid cell reactions. The renal tubular epithelium showed a series of degenerative changes ranging up to necrosis. The liver samples showed variable hepatic injury in the form of thickening in the Glisson capsule, as well as dissociation and disorganization of hepatic cords. Hepatocellular vacuolar degeneration, presence of focal areas of nodular hyperplasia, the hyperplastic cells mixed with lymphocytic infiltration, congestion in the portal vein, periportal fibrosis and edema with the presence of newly formed nonfunctional bile ductulus. Based on the histopathology scores, the severity of renal and hepatic changes was significantly increased (*P* ≤ 0.05) in all of the experimental groups compared with the control group. Generally, the chemical composition, microbiological analysis and vital organs were significantly affected by using cultured white soft cheese.

## Introduction

White cheese has been made in Egypt for a long time. Photographic evidence of Egyptian cheese making was found on the murals of ancient Egyptian tombs, which date back to nearly 20,000 years BC. In recent times, the production and consumption of white soft cheese have been increased because of it being an inexpensive source of protein and a rich source in minerals and vitamins as well as having a high nutritional value. Cheese is considered a good potential source of probiotic bacteria for the intestinal tracts of humans, which is related to its unique physical characteristics and chemical composition compared with fermented milk products (higher pH value and lower titratable acidity, higher total solid and fat content, and higher nutrient availability)^1^. The use of starter cultures containing lactic acid bacteria has become a requirement in the cheese industry, and its main function is the production of lactic acid and different flavor compounds, which results in different types of cheese^2^. The addition of a starter culture of lactic acid bacteria in cheese making has led to an improvement in the microbiological quality of the cheese besides a slight improvement in the physical, chemical and sensory properties of white cheese^3^.

The use of starters in the production of cheese leads to a relatively low pH and encourages the separation of whey. It also prevents the growth of pathogenic bacteria, besides the generation of some aroma compounds and increases the degree of maturity of cheese^4^. Lactobacilli possess several peptide-degrading enzymes, which can hydrolyze the peptides into oligo-peptides and amino acids which may alter the flavor, body, texture, and consequently the sensory properties of cheese^5^.

The kidneys and liver are considered vital organs involved in metabolism, detoxification, storage, and excretion and are particularly exposed to damage^6–8^. The kidneys and liver of rats fed with Domiati cheese showed several pathological effects^9,10^. No previous literature has studied the effect of cultured white soft cheese on the kidneys and liver of rats using *L. helveticus, L. rhamnosus* and *S. thermophilus* S_3855_.Thus, this study aims to investigate the possibility of making a cultured white soft cheese using *L. helveticus*, *L. rhamnosus,* and *S. thermophilus* S_3855_ and monitor the changes in the chemical composition and microbiological properties of cheese during the pickling period. Moreover, the impact of cultured white soft cheese on the histopathological changes in the kidneys and liver of experimental rats was evaluated.

## Materials and methods

Fresh cow’s milk was obtained from the Herd of the Animal Production Department, Faculty of Agriculture, Al-Azhar University (Branch of Assiut). An enzyme source (microbial rennet powder) was obtained from DSM (France) with a commercial name (Fromase R 2200). Salt: commercial sodium chloride was obtained from El-Nasr Company (Alexandria, Egypt). Starter strains: *Lactobacillus helveticus* (ATCC15009) was obtained from Cairo Microbiological Resource Center (MIRCEN), Faculty of Agriculture, Ain Shams University. *Lactobacillus rhamnosus* was obtained from the Dairy Department, Facultyof Agriculture, Al-Azhar University (Cairo). The *S. thermophilus* S_3855_ encapsulated strain was obtained from the Department of Dairy Science, Faculty of Agriculture Minia University. The albino rats were obtained from the Farm of National Organization for Drug Control and Research, Giza, Egypt.

### Manufacture of white soft cheese

White soft cheese was made by conventional method of making Domiati cheese according to the adopted method of^11^ with some modifications as follows:

Cow’s milk (3% fat) was divided into four equal portions. Every part was heated to 72 ± 1 °C for 15 min, and 4% salt (w/w) was added. It was then rapidly cooled to 40 °C and starters were added, followed by the addition of rennet for all portions:

**Table:** 

Control cheese	Nonstarter cheese
*L. helveticus* cheese	Adding 0.75% (w/w) *L. helveticus* starter
*L. rhamnosus* cheese	Adding 0.75% (w/w) *L. rhamnosus* starter
*S. thermophilus* S_3855_ cheese	Adding 0.75% (w/w) *S. thermophilus* (S_3855_) starter

Approximately 750 g of the resultant cheese was immersed in plastic containers (1000 g) filled up with cheese whey which was previously heated to 90 °C. The cheese containers were stored in a refrigerator at 6 ± 1 °C and the cheese samples were taken fresh and after 7, 14, 21 and 28 days for analysis.

### Chemical analysis

Moisture content and titratable acidity were determined using the IDF^12^ and AOAC^13^ methods, respectively. The pH values were measured using a pH meter (model 68 ESD 19713 USA). Fat content was determined using the AOAC methods^13^. Total nitrogen contents were determined as described using the IDF^14^.

### Microbiological analyses

The total bacterial count was determined using Marshall’s methods^15^. Lactobacilli count was estimated on a selective medium for lactobacilli (MRS) and streptococci count on an M17 agar medium, respectively^16^. Coliform bacteria were enumerated using the method of IDF^17^. Molds and Yeasts were enumerated in accordance with the FDA criteria^18^.

### Adaptation

The present study was performed according to the Egyptian laws and guidelines of the University for Animal Care. The Faculty of Veterinary Medicine National Ethical Committee, Assiut University, Egypt, has authorized all the steps in the present work. The study was reported in accordance with the ARRIVE guidelines (https://arriveguidelines.org).

The rats (Albino *Rattus norvegicus*) were housed in screen- bottomed aluminum cages in rooms maintained at 25 ± 1 °C, with a relative humidity of approximately 55% and given access to tap water for 28 days. The rats were fed with a basal diet for 1 week before starting the experiment. The ingredients of the basal diet were as follows: crude protein (12%), corn oil (8%), salt mixture (4%), vitamin mixture (1%), maize starch (70%) and fiber (5%).

### Experimental feeding techniques

Twenty-five male albino rats were randomly and equally divided into five groups, five rats each. After the acclimatization period for 7 days, each Group was fed with one of the following diets every day for 28 days.

**Table:** 

Group no	Diet
G1	Basal diet
G2	Basal diet (70%) + control cheese (30%)
G3	Basal diet (70%) + cheese containing *L. helveticus* (30%)
G4	Basal diet (70%) + cheese containing *L. rhamnosus* (30%)
G5	Basal diet (70%) + cheese containing *S. thermophilus S*_*3855*_ (30%)

### Specimen processing and staining

At the end of the respective experimental periods, the animals from each Group were weighted and then anesthetized via the intraperitoneal injection of equithensin (1 mL/kg). After the loss of all reflexes, the animals were transcardially perfused with warm saline followed by Trump’s fixative (3.7% formaldehyde plus 1% glutaraldehyde in saline buffer)^19^. Afterward, the kidney and liver specimens were obtained dissected and immediately fixed in 10% neutral buffer formalin for 24 h to ensure perfect fixation, after which they were dehydrated in a graded alcohol series, cleared in xylene, and finally embedded in paraffin. The tissue was cut into 3 μm thick sections and then stained with hematoxylin and eosin^20^. The sections were examined for histopathological changes using an OLYMPUS BX51 microscope and photographed with an OLYMPUSDP72 camera adapted to the microscope (Department of Anatomy and Histology, Assiut University, Egypt).

### Ordinal method for validating histopathologic scoring

Each animal was assigned a score on the basis of the tissue histopathological examination^21^. The samples were scored quantitatively and semiquantitatively, with an assessment based on the visual field inspection of a minimum of 10 sections from each Group. Histopathological lesions were scored by an experienced veterinary pathologist who was unaware of the experimental treatments or their information in advance. The photographs were taken at a magnification of 40 × and the cell numbers of the hepatocyte alterations were counted in 10 randomized areas (each 1 mm^2^)^22,23^^.^

Furthermore the severity of of each lesion was scored as follows: 0 = no lesions; 1 = minimal (1–10% of the tissue section affected); 2 = mild (11–25%); 3 = moderate (26–45%) and 4 = severe (> 45%)^21,22,24^_._

### Statistical analysis

Collected data were subjected to the analysis of variance (ANOVA) using the Statistical Analysis System at a 5% level of significance. The mean differences were separated using the least significant difference and expressed as means ± SE. The Shapiro -Wilk’s W test was conducted for the assumption of normality in which the test was insignificant.

Data on the histopathological results were expressed as mean ± S.D. The measurements obtained from the experimental groups were statistically estimated via GraphPad Prism (version 5) (San Diego, California, USA) using one‑way ANOVA with Tukey’s post hoc multiple comparison tests. A *P* value of *P* < 0.05 was considered statistically significant^22,24^.

## Result and discussion

Table 1 shows the data on the chemical composition of white soft cheese made with different strains of *L. helveticus*, *L. rhamnosus* and *S. thermophilus* S_3855_ during pickling periods, which was stored in a refrigerator for up to 28 days.

**Table 1:** Chemical composition of white soft cheese held at refrigerator temperatures up to 28 days.

Treatment	Storage periods	pH	Acidity %	Moisture %	Protein %	Fat/DM %
Control cheese	Fresh	6.43 ± 0.03 ^A^	0.17 ± 0.00 ^H^	64.32 ± 0.55 ^H^	11.87 ± 0.13 ^DEF^	29.16 ± 0.46 ^HIJ^
	7 days	6.25 ± 0.06 ^B^	0.20 ± 0.01 ^H^	65.10 ± 0.27 ^GH^	11.65 ± 0.21 ^DEF^	29.51 ± 0.22 ^FGHIJ^
	14 days	5.92 ± 0.03 ^D^	0.97 ± 0.02 ^F^	68.42 ± 0.18 ^DE^	11.27 ± 0.68 ^EF^	31.25 ± 0.23 ^ABCD^
	21 days	5.29 ± 0.03 ^HI^	1.43 ± 0.03 ^CD^	68.57 ± 0.55 ^CDE^	10.89 ± 0.97 ^EF^	31.09 ± 0.61 ^ABCD^
	28 days	5.21 ± 0.01 ^J^	1.50 ± 0.06 ^BC^	69.13 ± 0.12 ^CD^	10.46 ± 1.25 ^F^	31.64 ± 0.18 ^AB^
*L. helveticus* cheese	Fresh	6.46 ± 0.01 ^A^	0.15 ± 0.00 ^H^	63.99 ± 2.31 ^H^	13.95 ± 0.08 ^BC^	28.87 ± 1.33 ^IJ^
	7 days	6.08 ± 0.03 ^C^	0.21 ± 0.00 ^GH^	65.00 ± 0.24 ^GH^	15.31 ± 0.07 ^AB^	28.57 ± 0.24 ^J^
	14 days	5.58 ± 0.03 ^G^	1.13 ± 0.03 ^E^	67.97 ± 0.44 ^DEF^	13.87 ± 0.06 ^BC^	30.19 ± 0.36 ^CDEFGH^
	21 days	5.23 ± 0.02 ^IJ^	1.50 ± 0.06 ^BC^	72.08 ± 0.43 ^AB^	12.53 ± 0.11 ^CDE^	31.41 ± 0.27 ^ABC^
	28 days	5.19 ± 0.00 ^J^	1.57 ± 0.03 ^B^	72.75 ± 0.02 ^A^	11.53 ± 0.11 ^DEF^	32.18 ± 0.12 ^A^
*L. rhamnosus* cheese	Fresh	6.49 ± 0.02 ^A^	0.15 ± 0.00 ^H^	61.69 ± 0.21 ^I^	16.12 ± 0.08 ^A^	28.89 ± 0.23 ^IJ^
	7 days	5.68 ± 0.01 ^F^	0.29 ± 0.01 ^G^	67.67 ± 0.23 ^DEF^	14.16 ± 0.04 ^BC^	30.42 ± 0.16 ^BCDEFG^
	14 days	5.17 ± 0.04 ^JK^	0.93 ± 0.03 ^F^	66.45 ± 0.67 ^FG^	14.16 ± 0.06 ^BC^	30.03 ± 0.69 ^DEFGHI^
	21 days	5.10 ± 0.02 ^KL^	1.57 ± 0.03 ^B^	68.18 ± 0.43 ^DEF^	13.06 ± 0.06 ^CD^	31.02 ± 0.51 ^ABCDE^
	28 days	5.08 ± 0.01 ^L^	1.67 ± 0.03 ^A^	69.17 ± 0.03 ^CD^	11.29 ± 0.04 ^DEF^	31.57 ± 0.12 ^AB^
*S. thermophilus* S_3855_ cheese	Fresh	6.47 ± 0.01 ^A^	0.14 ± 0.00 ^H^	64.80 ± 0.81 ^GH^	15.46 ± 2.15 ^AB^	29.26 ± 0.04 ^GHIJ^
	7 days	6.29 ± 0.03 ^B^	0.18 ± 0.00 ^H^	67.08 ± 0.80 ^EF^	14.95 ± 0.08 ^AB^	29.80 ± 0.24 ^EFGHIJ^
	14 days	5.77 ± 0.01 ^E^	1.10 ± 0.06 ^E^	69.24 ± 0.41 ^CD^	14.27 ± 0.08 ^BC^	30.56 ± 0.10 ^BCDEF^
	21 days	5.32 ± 0.00 ^H^	1.40 ± 0.00 ^D^	70.46 ± 0.49 ^BC^	13.87 ± 0.04 ^BC^	30.68 ± 0.16 ^BCDEF^
	28 days	5.28 ± 0.01 ^HI^	1.53 ± 0.03 ^B^	71.75 ± 0.14 ^AB^	11.48 ± 0.04 ^DEF^	30.68 ± 0.20 ^BCDEF^

Means, in the same column, followed by the same letter are not significantly different at a probability level of 0.05.

### pH values

The data in Table 1 indicated the significant difference in the pH values of white soft cheese (*P* < 0.05) among all cheese treatments. The pH values of the control cheese, *L. helveticus* cheese, *L. rhamnosus* cheese and *S. thermophilus* S_3855_ cheese ranged from 6.43 ± 0.03 to 5.21 ± 0.01, 6.46 ± 0.01 to 5.19 ± 0.00, 6.49 ± 0.02 to 5.08 ± 0.01 and 6.47 ± 0.01 to 5.28 ± 0.01, respectively. The pH values of all cheese samples gradually decreased during the storage peroid of up to 28 days because of the accumulation of lactic acid produced by the lactic acid bacteria. These results are in consistent those reported by Perotti et al.^25^_,_ Mansour and Zaki^26^ and Zonoubi and Goli^27^.

### Titratable acidity (TA %)

The data in the same table indicated that the acidity of white soft cheese increased with the increase in storage periods at refrigerator temperatures up to 28 days. The control samples had lower TA than those of the other treatments at the end of the pickling period (28 days) as the control samples doesn’t inoculated with any strain. Conversely, the treatment containing the *L. rhamnosus* strain had the highest TA% at the end of the storage period. The differences TA titratable acidity among the cheese treatments might be attributed to the varying growth rates of microorganisms and their ability to ferment lactose during the pickling period. The same trend was obtained by Mansour and Zaki^26^, Zonoubi and Goli^27^ and Naeim^28^_._

### Moisture content

The data in Table 1 indicated that the moisture content of white soft cheese increaed with the increase in pickling periods at refrigerator temperatures up to 28 days. The treatment containing the *L. helveticus* strain and stored for 28 days had a higher moisture content than the other treatments. This increase of moisture content could be attributed to the refrigerator temperature which helps to imbibe the whey into the curd. Similar results were reported by Kebary et al.^5^.

### Protein content

White soft cheese showed a clearly significant (*P* < 0.05) decrease in protein content during the pickling period in all treatments. The decrease in protein content related to the increase in the moisture content of white soft cheese during the pickling period. However, the protein content of cheese samples was agreed with the results that found by^29–31^.

### Fat/dry matter (F/DM)

The data in the same table indicated that, the F/DM of white soft cheese increased with the increase in pickling periods at refrigerator temperatures up to 28 days in all treatments. Moreover, the cheese samples containing the *S. thermophilus* S_3855_ strain had a lower F/DM than the other cheese tsamples at at the end of the pickling period (28 days). Conversely, the treatment containing the *L. helveticus* strain had the highest F/DM at the end of the pickling period. Similar results were obtained by Kebary et al.^5^ and El-Sayed and El-Sayed^31^.

Table 2 shows the microbiological analyses of white soft cheese made with deferent strains of lactic acid bacteria (*L. helveticus*, *L. rhamnosus* and *S. thermophilus* S_3855_) and pickled at refrigerator temperatures up to 28 days.

**Table 2:** Microbiological analysis (log cfu/g) of white soft cheese held at refrigerator temperatures up to 28 days.

Treatments	Storage periods	Streptococci count	Lactobacilli count	Total count
Control cheese	Fresh	3.65 ± 0.03 ^L^	3.70 ± 0.10 ^M^	3.86 ± 0.01 ^N^
	7 days	4.18 ± 0.02 ^J^	4.05 ± 0.03 ^L^	4.35 ± 0.01 ^M^
	14 days	4.57 ± 0.02 ^I^	4.69 ± 0.01 ^K^	4.81 ± 0.01 ^L^
	21 days	4.59 ± 0.01 ^I^	5.13 ± 0.01 ^I^	5.19 ± 0.00 ^J^
	28 days	4.08 ± 0.04 ^K^	4.97 ± 0.02 ^J^	5.05 ± 0.02 ^K^
*L. helveticus* cheese	Fresh	5.36 ± 0.02 ^EFG^	8.42 ± 0.00 ^E^	8.50 ± 0.01 ^HI^
	7 days	5.40 ± 0.01 ^DEF^	8.48 ± 0.00 ^CDE^	8.67 ± 0.03 ^D^
	14 days	5.44 ± 0.01 ^D^	8.54 ± 0.01 ^BC^	8.75 ± 0.01 ^BC^
	21 days	5.37 ± 0.01 ^EF^	8.53 ± 0.02 ^C^	8.56 ± 0.01 ^FG^
	28 days	5.28 ± 0.01 ^H^	8.50 ± 0.01 ^CD^	8.50 ± 0.01 ^H^
*L. rhamnosus* cheese	Fresh	5.35 ± 0.01 ^FG^	8.44 ± 0.01 ^DE^	8.54 ± 0.01 ^GH^
	7 days	5.40 ± 0.00 ^DEF^	8.61 ± 0.01 ^AB^	8.74 ± 0.02 ^C^
	14 days	5.41 ± 0.00 ^DE^	8.67 ± 0.01 ^A^	8.82 ± 0.01 ^A^
	21 days	5.36 ± 0.01 ^EFG^	8.62 ± 0.01 ^AB^	8.62 ± 0.01 ^E^
	28 days	5.32 ± 0.01 ^GH^	8.60 ± 0.01 ^AB^	8.60 ± 0.01 ^EF^
*S. thermophilus* S_3855_ cheese	Fresh	8.50 ± 0.01 ^B^	5.27 ± 0.02 ^H^	8.67 ± 0.03 ^D^
	7 days	8.67 ± 0.01 ^A^	5.48 ± 0.01 ^F^	8.83 ± 0.03 ^A^
	14 days	8.54 ± 0.03 ^B^	5.45 ± 0.01 ^F^	8.78 ± 0.01 ^AB^
	21 days	8.50 ± 0.02 ^B^	5.43 ± 0.01 ^FG^	8.55 ± 0.01 ^G^
	28 days	8.43 ± 0.01 ^C^	5.37 ± 0.01 ^G^	8.46 ± 0.00 ^I^

Means, in the same column, followed by the same letter are not significantly different at a probability level of 0.05.

### Streptococci counts

Significant differences (*P* < 0.05) in streptococci count (Tale 2) were observed, and the count ranged from 3.65 ± 0.03 to 8.67 ± 0.01 log cfu/g^-1^. Moreover, it was indicated that the control samples had lower streptococci counts than other cheese samples. Conversely, the samples of *S. thermophilus* S_3855_ cheese had the highest streptococci counts (8.67 ± 0.01 log cfu g^-1^) at 7 days of the pickling period. The streptococci counts of *L. helveticus* cheese and *L. rhamnosus* cheese were higher than that of the control cheese. This may be related to the synergistic effect between lactobacilli and streptococci during ripening. Moreover, the streptococci counts of were increased in the beginning days during the storage period and then decreased up to the end of pickling period. These results are consistent with those obtained by Papademas et al.^32^ and Dafalla and Abdel Razig^33^.

### Lactobacilli count

The obtained data in the same table indicated that the treatment of *L. helveticus* cheese and *L. rhamnosus* cheese had higher lactobacilli counts than the control and *S. thermophilus* S_3855_ cheese. Moreover, the lactobacilli counts were increased with the increase in storage periods to reach the highest count in 14 days of the pickling period in all cheeses. For the except S_3855_ cheese, if the highest count was reached in 7 days, then the counts were decreased in all treatments up to the end of the pickling period. Moreover, the data of lactobacilli counts indicated significant differences (*P* < 0.05) among all treatments, the count ranging from 3.70 ± 0.10 to 8.67 ± 0.01 log cfu g^−1^^34–36^_._

### Total bacterial count

Table 2 showed significant differences (*P* < 0.05) and indicated that the control samples had lower total bacterial counts than the other cheeses. Moreover, the highest counts were obtained from the *S. thermophilus* S_3855_ cheese samples that were pickled for 7 days. By contrast the lowest total counts were obtained from the fresh control samples. Furthermore, the total bacterial counts were increased at the beginning of the pickling period and then decrease up to 28 days in all treatments. These results are agreed with those of Kebary et al.^5^ and Dafalla and Abdel Razig^33^.

**Figure 1:**
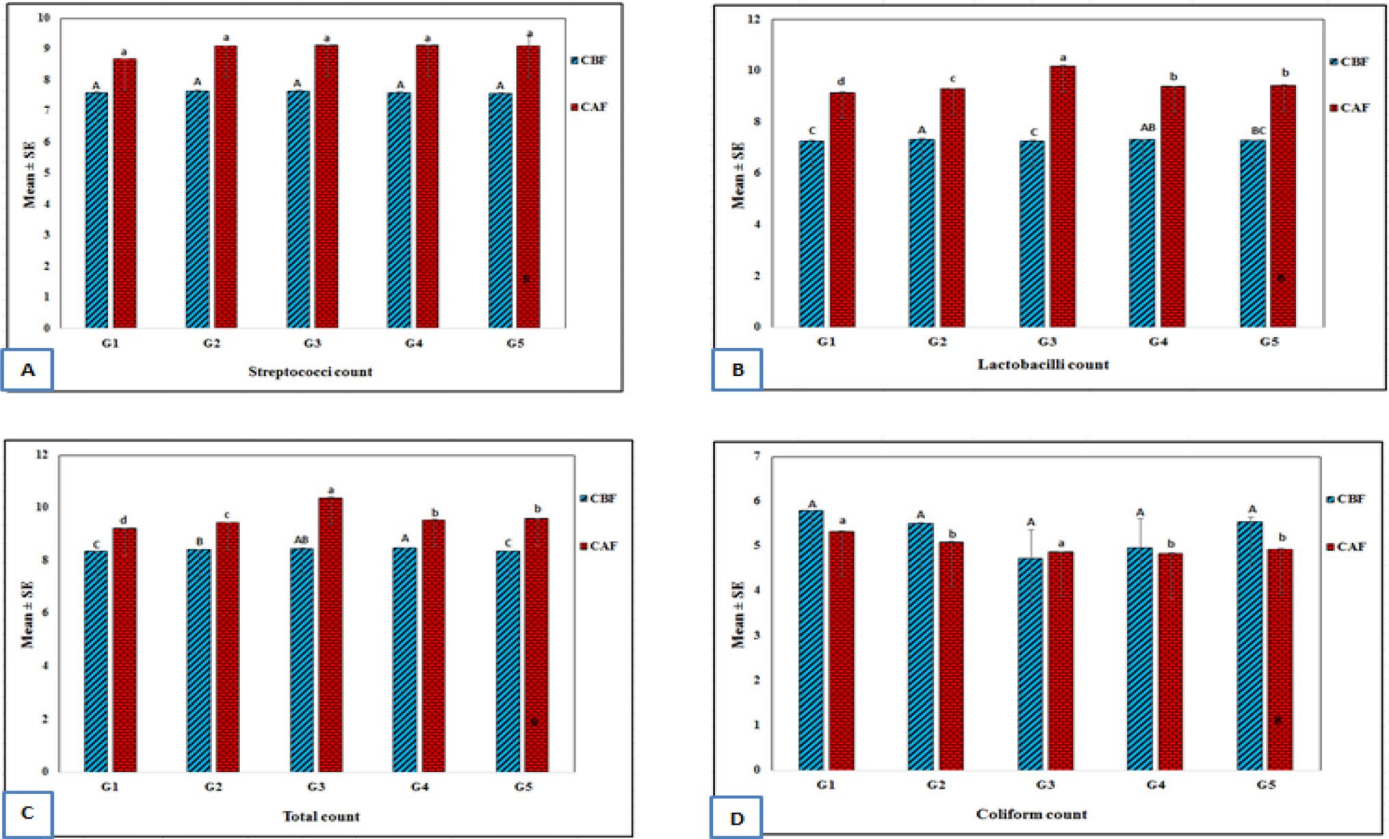
Bacteriological properties (log cfu/g feces) of albino rat feces before and after feeding with white soft cheese. (**A**) Streptococci count, (**B**) Lactobacilli count, (**C**) Total bacterial count, and (**D**) Coliform counts. CBF, counts in feces before feeding; CAF, counts in feces after feeding.

Figure 1 shows the bacteriological properties (streptococci count, lactobacilli count, total bacterial count and coliform count) of rat feces before and after feeding with white soft cheese.

### Streptococci count

The obtained data in Fig. 1A indicated that, the streptococci counts before and after feeding with white soft cheese did not show any significant difference (*P* > 0.5) . In all rat groupd, it is clear that the streptococci counts after feeding with white soft cheese were higher than those before feeding due to the increase in the number of streptococci in inoculated and uninoculated cheese. These results are similar with those obtained by^37^.

### Lactobacilli count

The obtained data in Fig. 1B indicated that, the lactobacilli counts before and after feeding with white soft cheese among all rat groups showed significant difference (*P* < 0.05). Also, lactobacilli counts after feeding with white soft cheese were higher than those before feeding in all rat groups. Furthermore, Group 2 had the highest lactobacilli count before feeding with white soft cheese, whereas Group 3 had the highest lactobacilli counts after feeding.

### Total bacterial count

The obtained data in Fig. 1C showed significant differences (*P* < 0.05) and indicated that the treatment of Group 1 initialy had lower total bacterial count than that of the other expermintal groups, whereas , the treatment of Group 4 had the highest count. Conversly, the treatment of Group 1 after feeding had lower total bacterial counts than that of the other treatments whereas the treatment of Group 3 had the highest count.

### Coliform count

The obtained data in Fig. 1D indicated that the in treatment Group 3 initialy had the lowest coliform counts than that of the other treatments, whereas the treatment of Group 1 had the highest counts. Conversely, the treatment of Group 4 after 4 weeks of feeding had the lowest counts of coliform groups than that of the other treatments, whereas the treatment of Group 1 had the highest count. The data also indicated that the coliform count decreased after the feeding period in all groups, which could be attributed to the presence of lactic acid bacteria.These results are in agreement with those obtained by^38^, who found that the probiotic strain *L. plantarum* as a single species or in combination with oil (Lini oleum virginale) decreased the total coliform count of and increased the lactobacilli in the feces of rats.

### Histopathological observations

#### Renal histopathological changes

**Figure 2:**
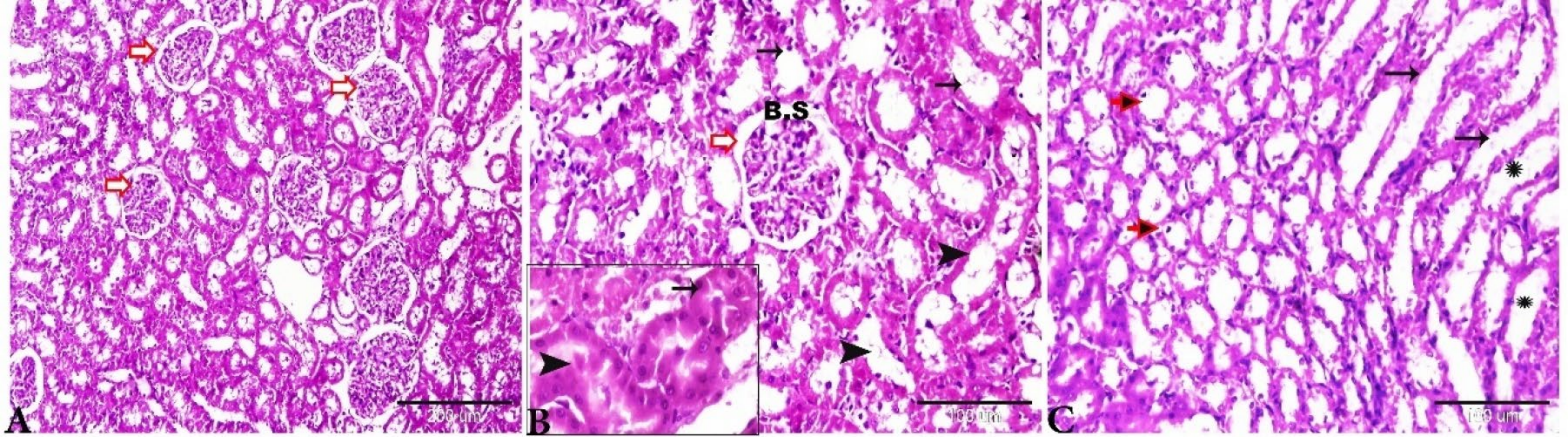
Photomicrograph of the rat kidneys of Group I showed normal histology architectures. (**A**, **B**) Renal cortex contains normal glomerulus (red arrows) and Bowman’s space (BS). Proximal convoluted tubules (B, thin arrows) and distal convoluted tubules (B, thick arrowheads), magnified in the selected square. (**C**) Normal renal medulla, thick ascending, Henle’s loop (black thin arrows), thick descending, Henle’s loop (stars), thin segment, Henle’s loop (red arrows). H&E stain, the bar size was indicated under pictures.

The histopathological changes in the liver tissue have been observed in all experimental groups apart from the control rats on the basis of the typical histological architecture of the normal renal parenchyma (Fig. 2) as observed previously by Brzóska et al.^39^.

**Figure 3:**
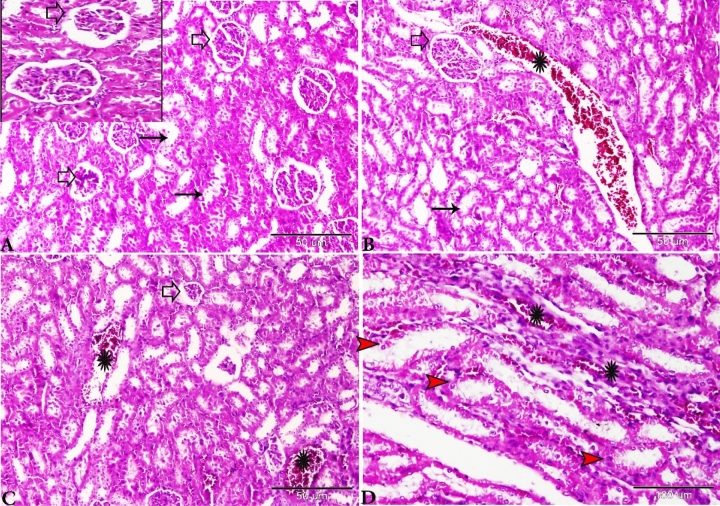
Photomicrograph of the rat kidneys from Group II (treated with basal diet 70% + control cheese 30%). (**A**–**C**) Glomerular atrophy (thick arrows, maximized in the selected square), degeneration and desquamation of the renal tubular epithelium (thin arrows), and cortical vessels widely dilated and congested (star), (**D**) Renal medulla showing the degeneration of renal tubules (Henle’s loop) (red arrowheads), vascular congestion (stars), and hemorrhage between the renal tubules. H&E stain, the bar size was indicated under pictures.

However, the sections taken from the rat kidneys of Group II (treated with basal diet 70% + control cheese 30%) showed several pathological findings: the cortical area showed glomerular atrophy, and the glomerular tuft was condensed and BS appeared to be wider than normal (Fig. 3A–C). Proximal and distal convoluted tubules exhibited degenerated epithelial lining. The vasculatures at the cortical area showed severe congestion; the blood vessels were dilated widely and engorged with blood (Fig. 3B,C). The tubules in the renal medulla showed vacuolation, degeneration and desquamation of its lining epithelium (Fig. 3D), and intense congestion was oserved in the blood vessels between the renal tubules (Fig. 3D).

**Figure 4:**
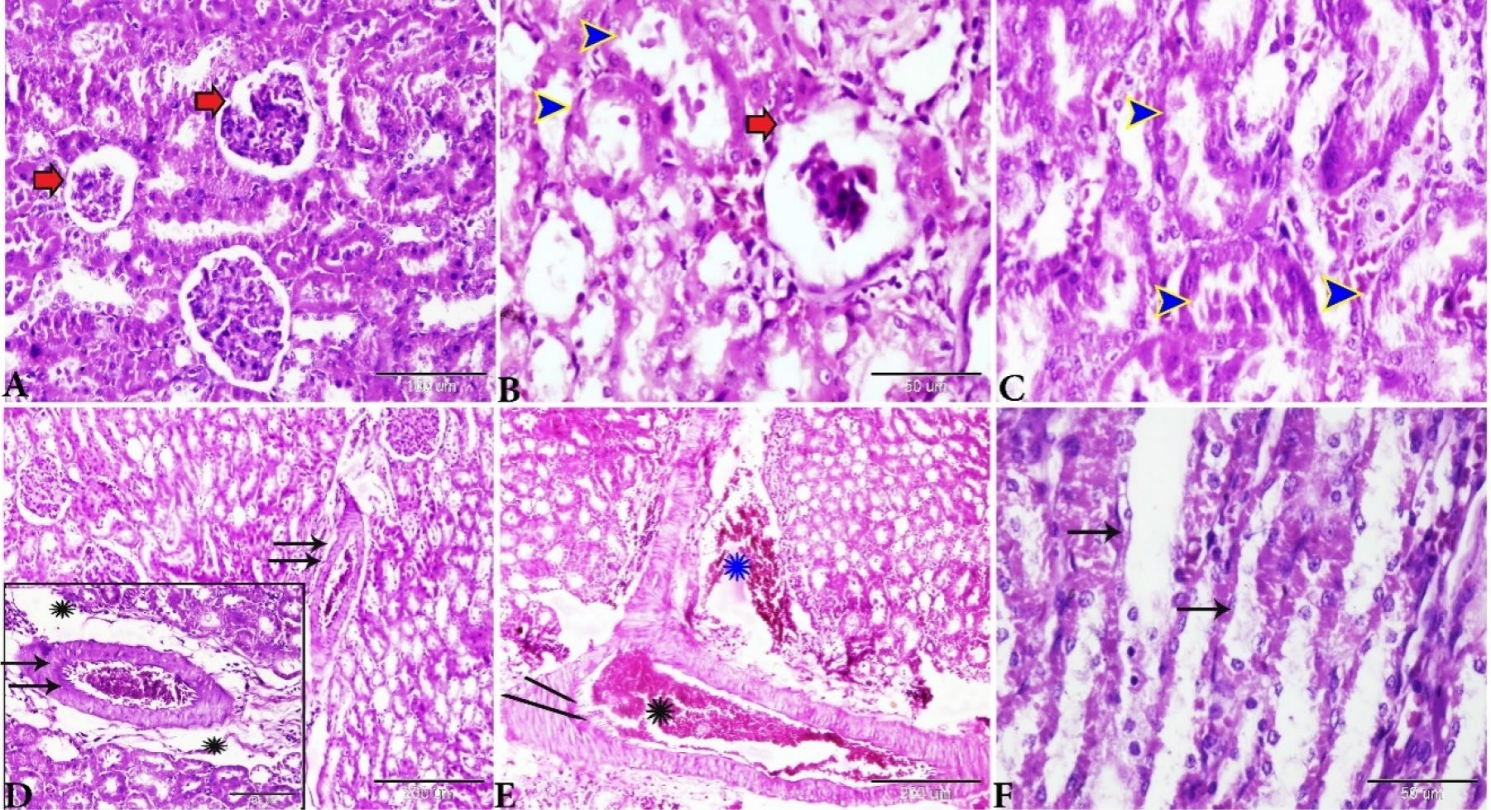
Photomicrograph of the rat kidneys of Group III (treated with basal diet 70% + cheese containing *L. helveticus* 30%). (**A**) Glomerular atrophy (red arrows). (**B**) Some areas showing severe atrophy and loss of glomerulus with degenerated renal corpuscle (red arrow). (**B**, **C**) Renal tubular degeneration and desquamation of its epithelial lining (blue arrows). (**D**) The arcuate artery at corticomedullary junction showing congestion (arrows) with perivascular edema (stars). (**E**) The arcuate artery showing severe congestion (black star), thick degenerated wall (scales), perivascular hemorrhage and edema (blue star). (**F**) Renal medulla tubules showing severe degeneration and desquamation of its lining epithelium inside its lumen (arrows) with interstitial hemorrhage between the renal tubules. H&E stain, the bar size was indicated under pictures.

The renal sections of Group III rats (treated with basal diet 70% + cheese containning *L. helveticus* 30%) showed glomerular atrophy, and some renal corpuscles revealed a variable degree of glomerular loss (Figs. 4A,B). Degeneration of proximal and distal convoluted tubules was observed and the epithelium was desquamated inside the lumen of some tubules and appeared as epithelial casts (Fig. 4B,C). The arcuate artery located at the corticomedullary junction was dilated and congested with thickening of its wall due to fibroses. Perivascular edema and hemorrhage around the arcuate artery (Fig. 4D,E). Henle’s loop showed dilatation with desquamated epithelium inside its lumen (Fig. 4F).

**Figure 5:**
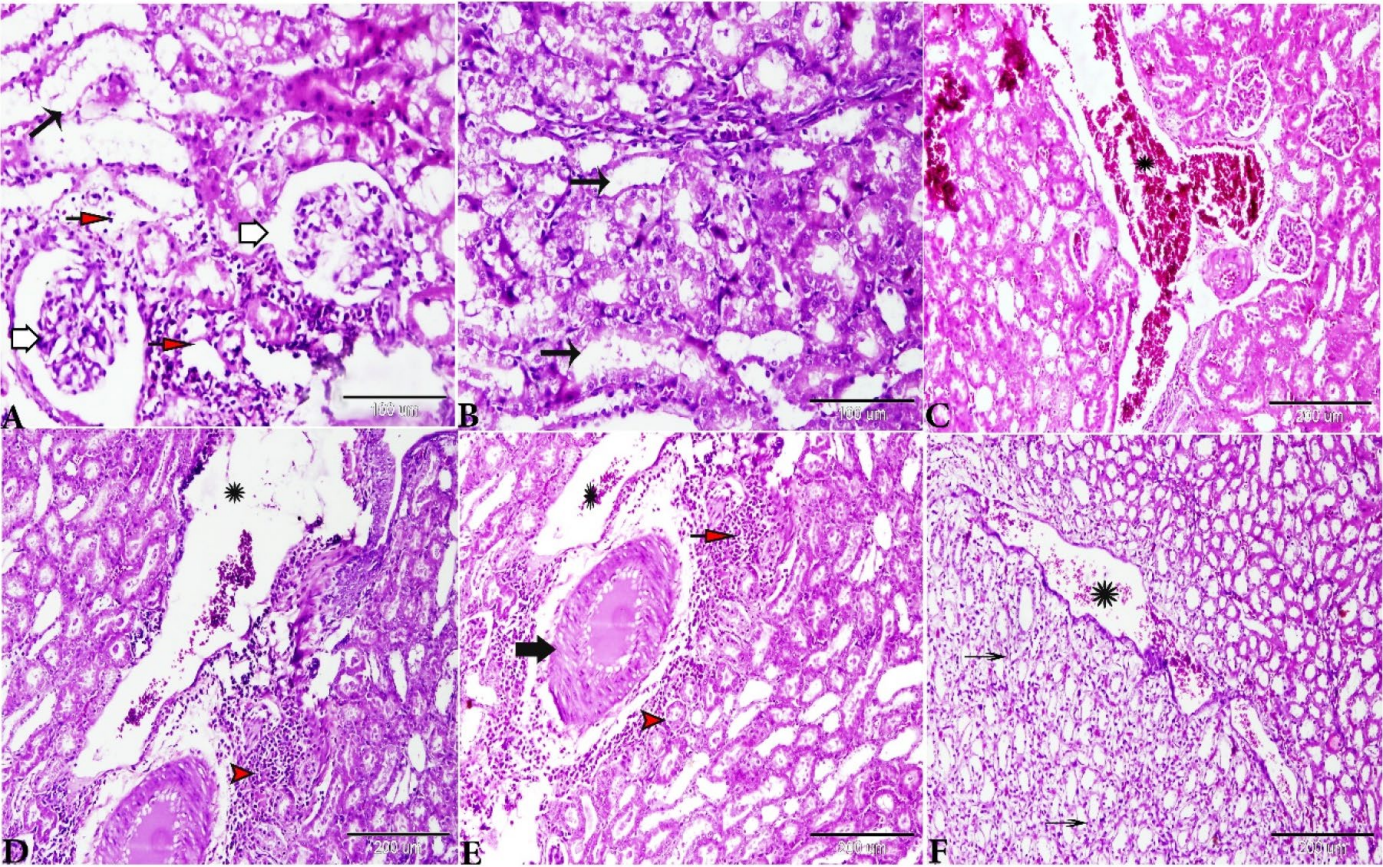
Photomicrograph of the rat kidneys of Group IV (basal diet 70% + cheese containing *L. rhamnosus* 30%). (**A**) Glomerular atrophy; BS widened and glomerulus showing necrobiotic changes (white arrows), renal tubules dilated with degeneration and necrosis of its epithelial lining (black arrow), interstitial mononuclear cellular infiltration (red arrows). (**B**) Disorganization and degeneration of renal tubules and vacuolation of the lining epithelium (black arrows). (**C**,**D**) Severe vascular dilatation and congestion (stars). Focal areas of mononuclear cellular aggregations (c, red head arrows). (**E**, **F**) Severe vascular dilatation and congestion in the corticomedullary junction (stars), fibrinoid degeneration of vascular wall (black arrow), severe perivascular focal mononuclear cellular aggregations (red arrow). (**F**) Degeneration in the renal medullary epithelium (arrows). H&E stain, the bar size was indicated under pictures.

The histopathological examination of the kidneys of Group IV (treated with basal diet 70% + cheese containing *L. rhamnosus* 30%) showed that the changes of the glomeruli comprise atrophy, distortion, and hypocellularity of the glomerular tuft. BS was widened and the nucleus of the remaining capillary tuft was condensed (Fig. 5A). The renal tubular epithelium showed a series of degenerative changes up to necrosis; both proximal and distal convoluted tubules were affected. The tubular epithelium sometimes showed the lysis of the cytoplasm and pyknosis of the nucleus. Sometimes the necrotic epithelium sloughed and the tubules were dilated. Changes in the tubules were diffuse and involved a massive area of the both cortex (Fig. 5B) and medulla (Fig. 5F). The most prominent histopathologic changes were present mainly in the renal vasculature of the glomeruli and renal tubules. The primary changes were present in the renal vasculature, especially those in the cortical area (Fig. 5C). The interstitial capillaries were dilated and engorged with blood. In some cases, the blood vessels at the corticomedullary junction were congested with a very mild perivascular edema (Fig. 5D,E). Focal small and multiple areas of lymphoid cell reactions were oserved in the interstitial areas and around the vasculatures (Fig. 5D,E). Our results are consistent with those of Al-Mathkhury and Baraaj^40^ who found that the renal tissues of the rats that were injected intraperitoneally with *Lactobacillus bulgaricus, Lactobacillu plantarum* and *Lactobacillu acidophilus* showed congestion of the vessels with blood and hemorrhage.

**Figure 6:**
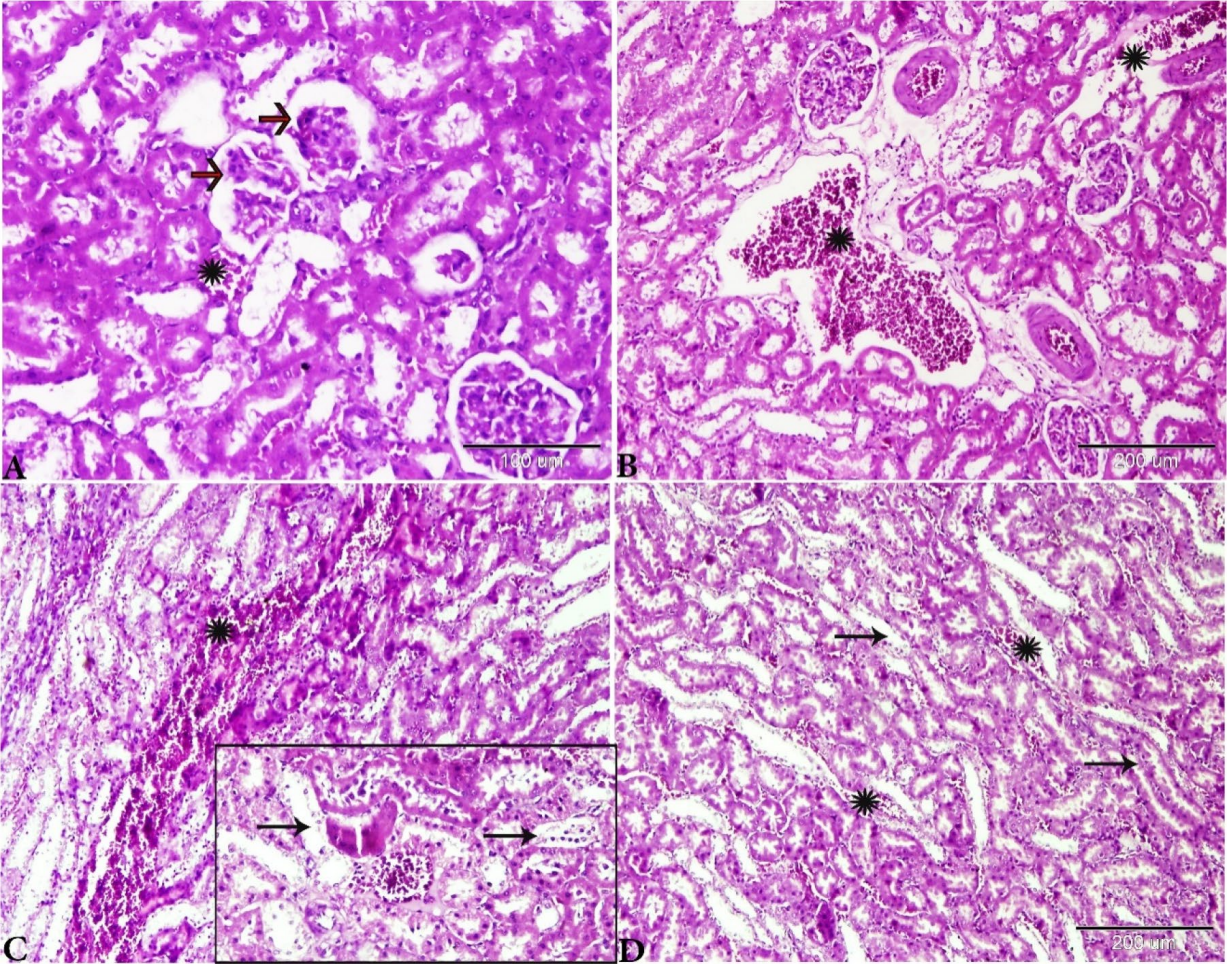
Photomicrograph of the rat kidneys of Group V (basal diet 70% + cheese containing *S. thermophilus* S_3855_ 30%), (**A**) glomerular atrophy (red arrows), interstitial hemorrhage (star). (**B**) Vascular congestion (stars). (**C**) Renal tubular necrosis (selected square, arrows), severe hemorrhage at the cortico-medullary junction (star). (**D**) Loop of Henle’s showing dilatation and desquamation of its lining epithelium (arrows) hemorrhage between the renal tubules (stars). H&E stain, the bar size was indicated under pictures.

The kidneys of Group V (treated with basal diet 70% + cheese containing *S. thermophilus* S_3855_ 30%) showed prominently glomerular atrophy and hypocellularity, which were associated with the widening the BS (Fig. 6A). Sever cortical vascular congestion (Fig. 6B), Severe necrotic changes of renal tubular epithelium in both cortex (Fig. 6C) and medulla (Fig. 6D); the necrotic changes were severe and diffuse , which resulted in the loss of the entire epithelium. Hemorrhage at the corticomedullary junction (Fig. 6C). The intertubular blood vessels were congested (Fig. 6D).

The kidneys of rats that were fed with Domiati cheese stored in a refrigerator exhibited regions of hemorrhage and edema in the tubules and in the interstitial areas. Moreover, it showed the degeneration of renal tubules; and partly the degeneration of renal corpuscles^9,10^.

**Figure 7:**
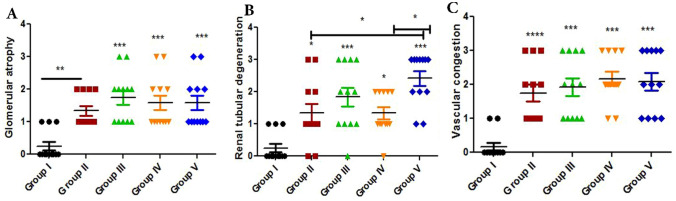
Histomorphometry graph showing the semiquantitaive measurements of rats kidney alterations between the experimental groups. Plots showing (**A**) glomerular atrophy, (**B**) renal tubular degeneration and (**C**) vascular congestion among the experimental groups. Data were represented as mean ± standard error. Significant differences versus the control groups determined through one-way ANOVA with Tukey’s post hoc test are marked by different asterisks: *P ≤ 0.05, **P ≤ 0.01, ***P ≤ 0.001).

Based on the histopathology scores, the severity of glomerular atrophy (Fig. 7A), renal tubular degeneration (Fig. 7B) and vascular congestion (Fig. 7C) were significantly increased (*P* ≤ 0.05) in all treated experimental groups compared with the control Group.

### Hepatic histopathological changes

**Figure 8:**
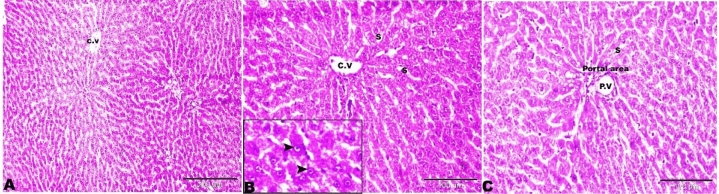
Photomicrograph of the rat liver Group I showed normal histological architectures. (**A**) Intact parenchymal hepatic architecture. (**B**) Normal central vein (C.V), hepatic sinusoids between hepatic lobules (S), hepatocytes (selected square, arrowheads). (**C**) Portal area containing normal portal veins (PV), hepatic artery, and bile duct. H&E stain, the bar size was indicated under pictures.

The microscopic examination of the rat liver of Group I revealed normal hepatic architectures which comprised, normal central vein, hepatic cords, hepatocytes and portal area contents (bile duct, hepatic artery, and vein) (Fig. 8).

**Figure 9:**
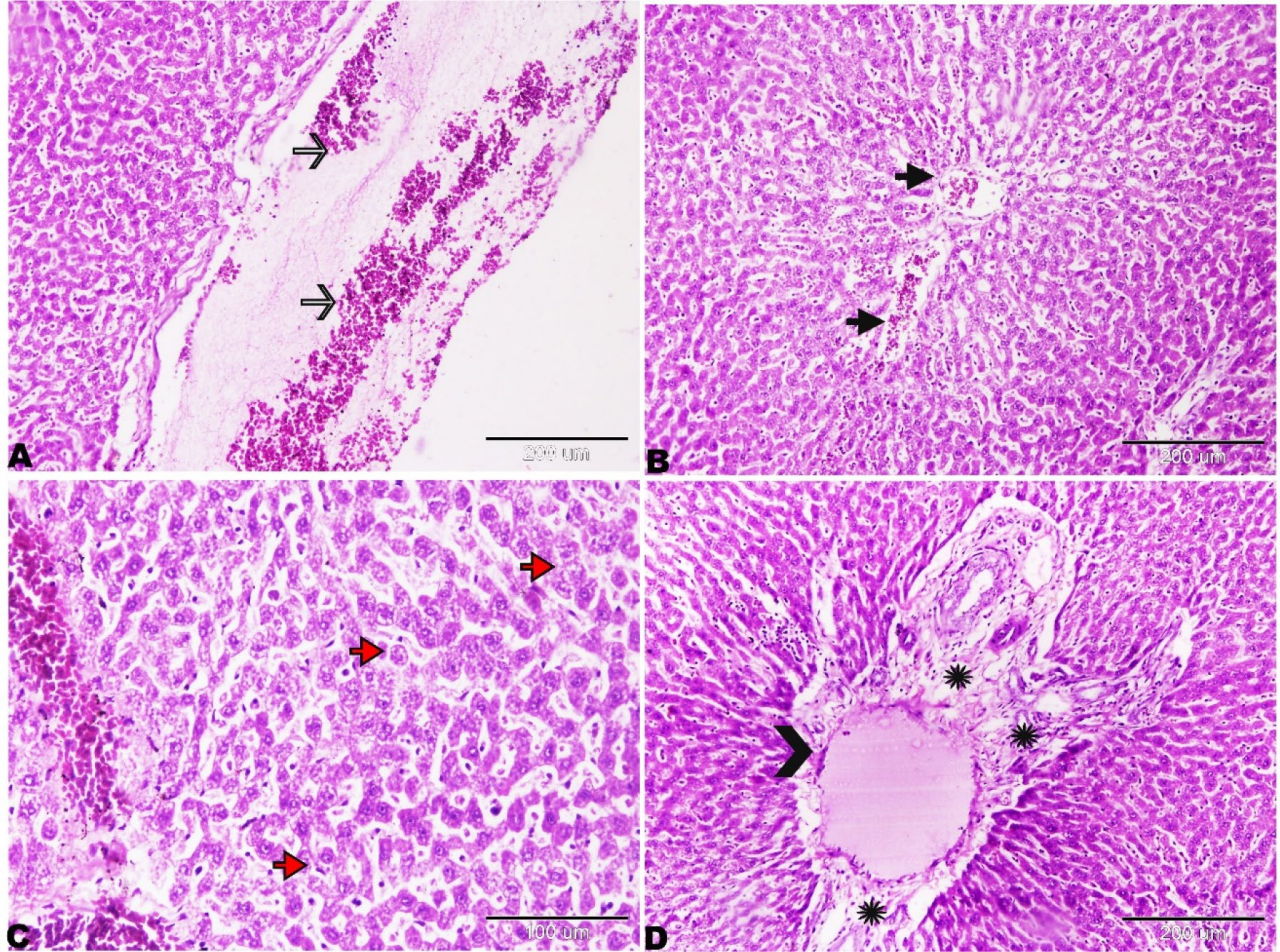
Photomicrograph of rat liver of Group II (treated with basal diet 70% + control cheese 30%). (**A**) Glisson capsule surrounding the liver was thickened and exhibits severe hemorrhage and edema (arrows). (**B**) Central vein was dilated and engorged with blood (arrows). (**C**) Dissociation of hepatic cords and vacuolation of hepatocytes (red arrows). (**D**) The portal areas showing dilated and congested portal veins (black arrowheads), periportal edema mixed with fine threads of fibrosis (stars). H&E stain, the bar size was indicated under pictures.

Liver sections taken from the rats kidneys of Group II (treated with basal diet 70% + control cheese 30%) showed variable hepatic injuries such asthickening in the Glisson capsule surrounding the liver with extensive hemorrhage and edema (Fig. 9A). Central vein dilated and engorged with blood (Fig. 9B), dissociation, and disorganization of hepatic cords, and vacuolation of hepatocytes (Fig. 9C). The portal areas involved wide areas in hepatic parenchyma as the portal veins extensively were dilated and filled with blood with periportal edema mixed with fine threads of fibrosis which branched in-between hepatic tissue (Fig. 9D).

**Figure 10:**
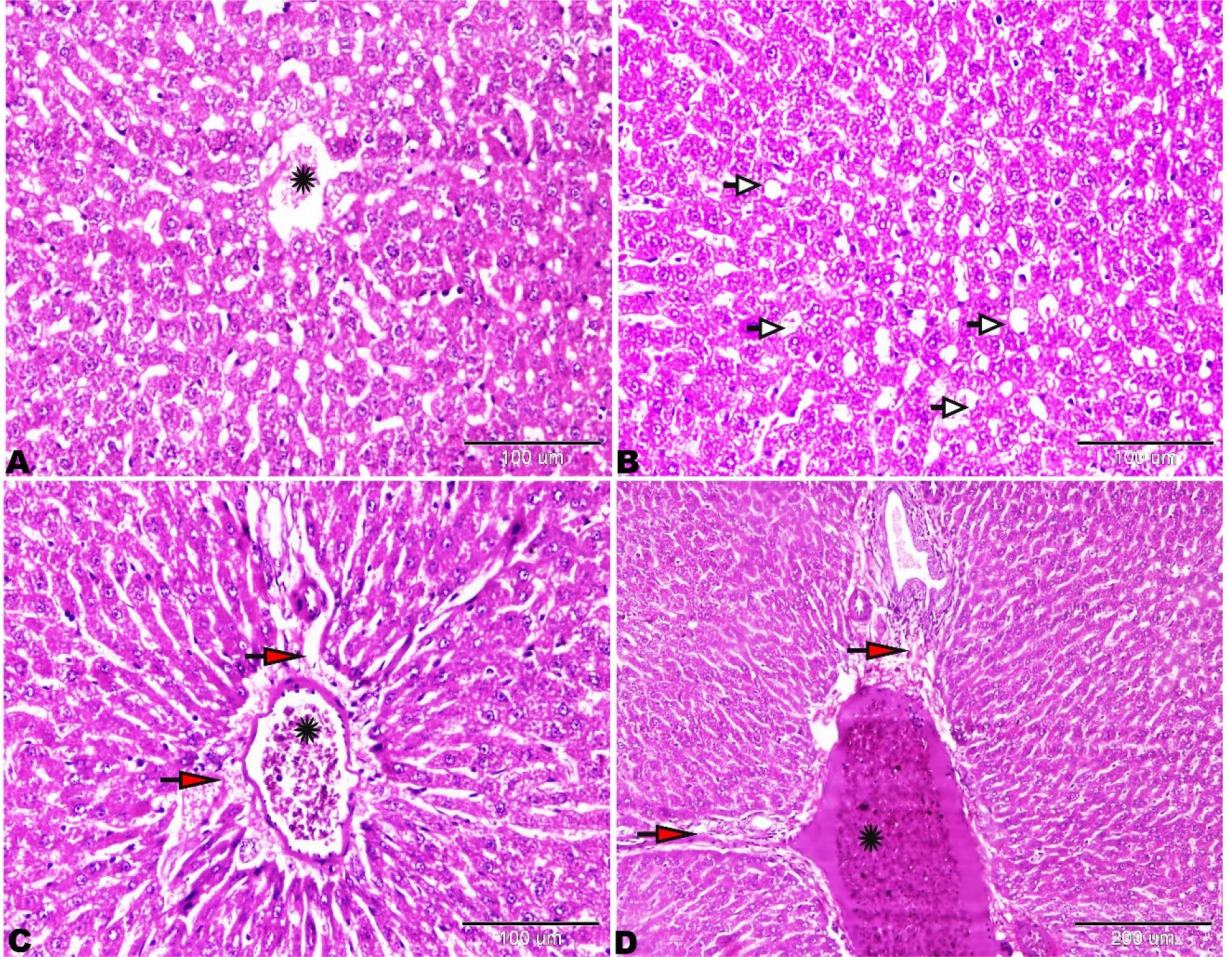
Photomicrograph of the rat livers of Group III (treated with basal diet 70% + cheese containing *L. helveticus* 30%). (**A**) Degeneration of the endothelium lining the central vein (star). (**B**) Vacuolar degeneration of hepatocytes (white arrows). (**C**, **D**) Portal area showing congestion in the portal veins with mononuclear inflammatory cellular infiltration (star), periportal fibroses (red arrows). H&E stain, the bar size was indicated under pictures.

The liver sections of Group III (treated with basal diet 70% + cheese containing *L. helveticus* 30%) showed congested central veins with degeneration and desquamation of its endothelium (Fig. 10A). Hepatocellular vacuolar degeneration, hepatic cells appearing as empty spaces with a marginally located flattened nucleus which similar to the appearance of signet rings (Fig. 10B). The portal areas showed congestionin the portal vein with severe periportal fibroses which extends as tracts between hepatic lobules (Fig. 10C,D).

**Figure 11:**
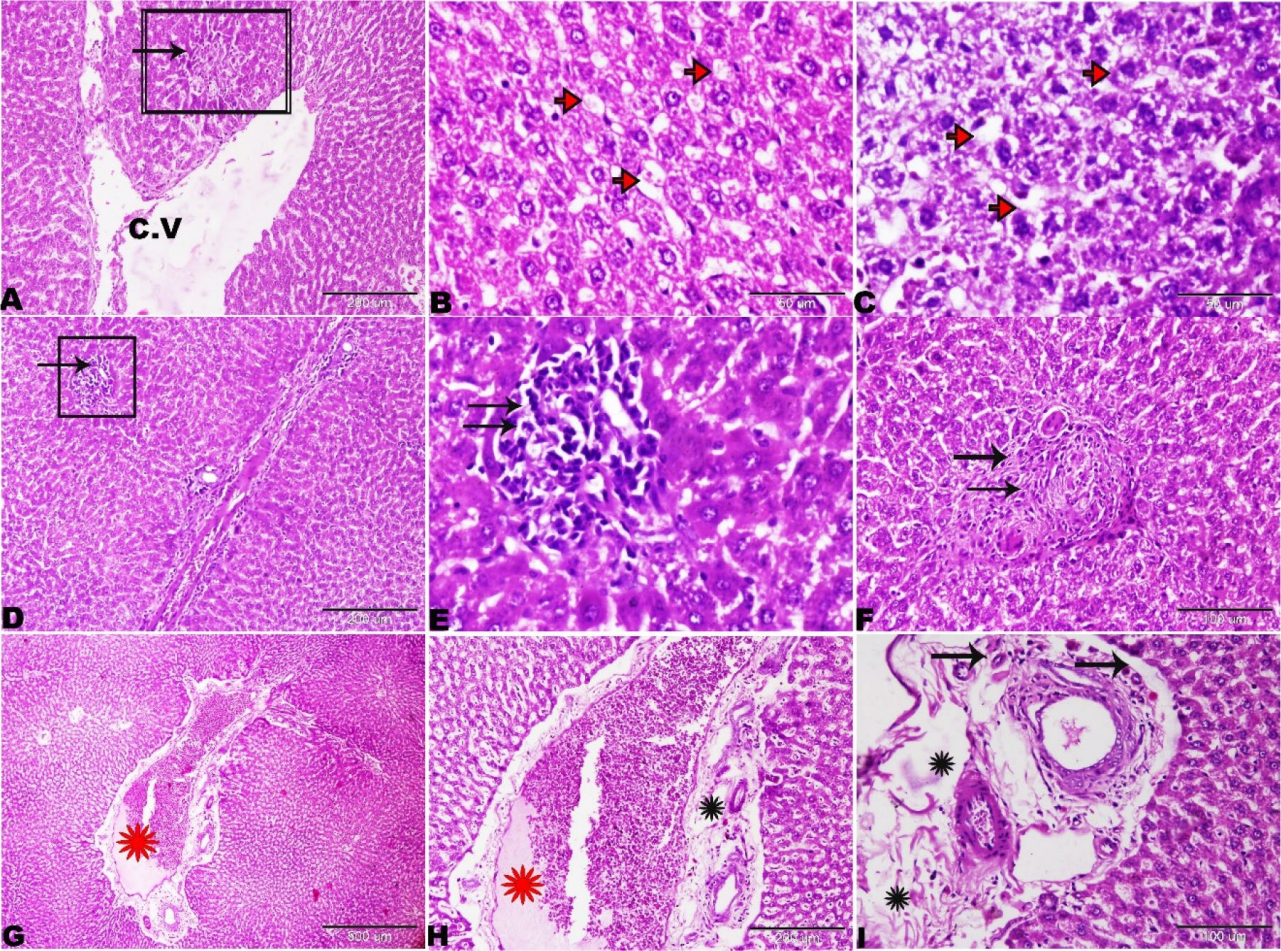
Photomicrograph of the rat livers of Group IV (basal diet 70% + cheese containing *L. rhamnosus* 30%). (**A**) Dilated central vein with degenerated endothelial lining (star), focal nodular hyperplasia (selected square). (**B**,**C**) Vacuolar degeneration of hepatocytes (red arrows). (**D**) Focal nodular hyperplasia (selected square) magnified in E. hyperplastic cells mixed with lymphocytic infiltration (arrows). (**F**) Focal nodular hyperplasia of the liver parenchyma and stellate scar with abnormal blood vessels. Part of the vessels showing a marked thickening of the wall without lumen (arrows). (**G**,**H**) Wide portal areas due to extensive widening and congestion in the portal veins (red star) and periportal edema (black star). I: periportal fibrosis and edema (stars) and newly formed bile ductulus (arrows). H&E stain, the bar size was indicated under pictures.

The histopathological examination of the rat liver of Group IV(treated with basal diet 70% + cheese containg *L. rhamnosus* 30%) showed dilated central vein with degenerated endothelial lining (Fig. 11A) and vacuolar degeneration of hepatocytes (steatotic like cells) (Fig. 11B,C). The presence of focal areas of nodular hyperplasia, the hyperplastic cells mixed with lymphocytic infiltration (Fig. 11D,E), other focal nodular areas exhibiting stellate scar with abnormal blood vessels, the vessels showing a marked thickening of the wall without lumen (Fig. 11A,F). Wide portal areas due to extensive widening and congestion in the portal veins with periportal fibrosis and edema, and the presence of newly formed nonfunctional bile ductulus (Fig. 11G–I). Similar results were obtained by^39^ who found that the liver tissues of the rats that were injected intraperitoneally with *L. bulgaricus*, *L. plantarum* and *L. acidophilus* showed inflammatory cells infiltration and also necrosis.

**Figure 12:**
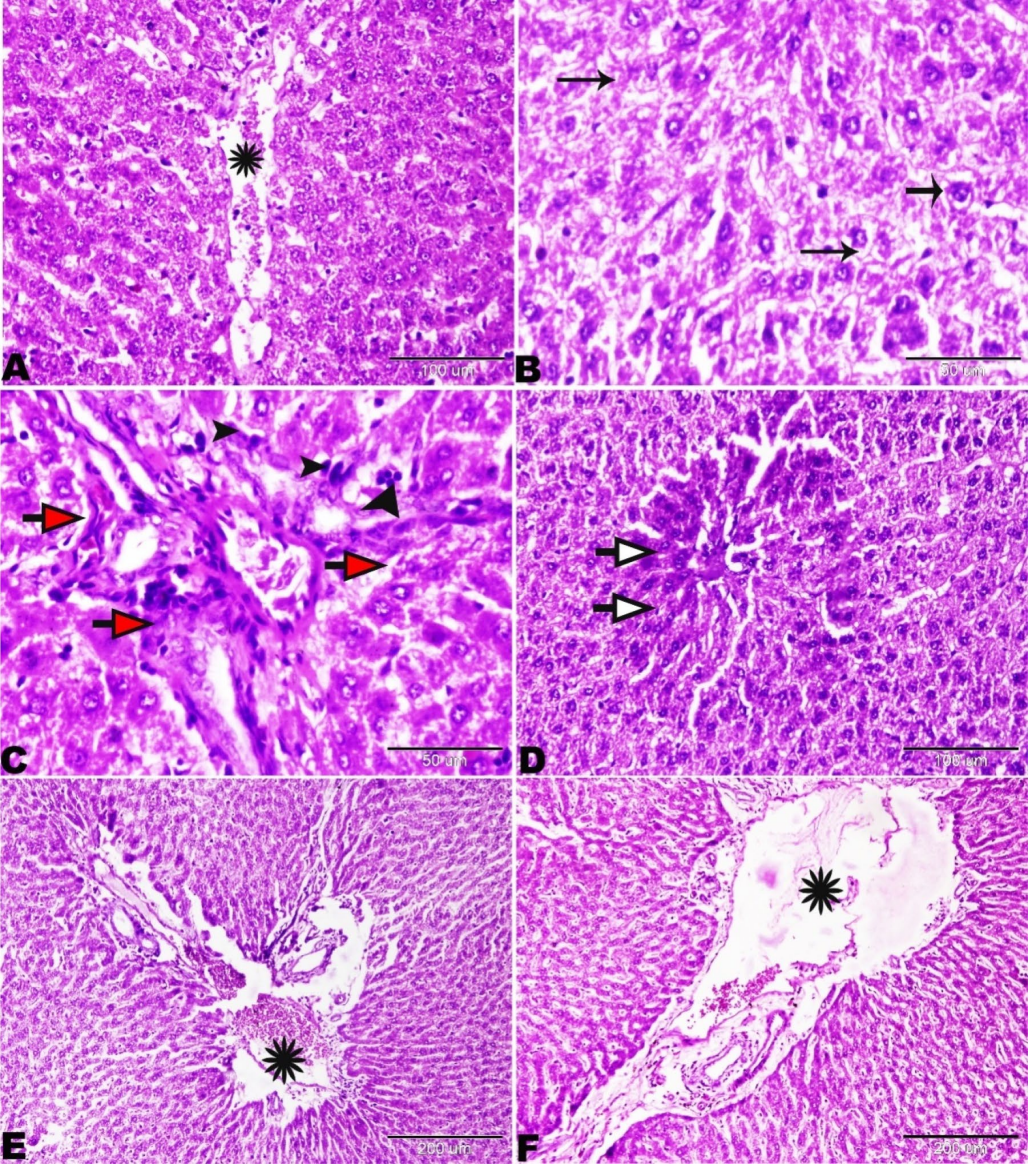
Photomicrograph of the rat livers of Group V (basal diet 70% + cheese containing S*. thermophilus* S_3855_ 30%). (**A**) Dilated central vein engorged with blood (star). (**B**) Vacuolation in hepatocytes (arrows). (**C**) Focal areas of fibroses (red arrows), mononuclear lymphocytic infiltration (black arrowheads). (**D**) Focal areas of coagulative necrosis (white arrows). (**E**) Wide portal areas showing extensively wide portal veins filled with blood (stars). (**F**) Severe periportal edema (star). H&E stain, the bar size was indicated under pictures.

The liver sections of Group V (basal diet 70% + cheese contaning *S. thermophilus* S_3855_ 30%) showing congested central vein (Fig. 12A), and vacuolation in hepatocytes (Fig. 12B). The focal areas of fibrosis with mild mononuclear lymphocytic infiltration (Fig. 12C). The focal areas of coagulative necrosis were also found (Fig. 12D). Wide portal areas showed extensively wide portal veins filled with blood with severe periportal edema (Fig. 12E,F).

The liver of rats fed with Domiati cheese stored in a refrigerator revealed vacuolation in the cytoplasm of the hepatocytes. Otherwise, the liver of the other rats showed the normal structure of the hepatic lobules. The histopathological changes that happened in the liver of rats were perhaps due to an increase in the levels of the biogenic amines of Domiati cheese^9,10^.

**Figure 13:**
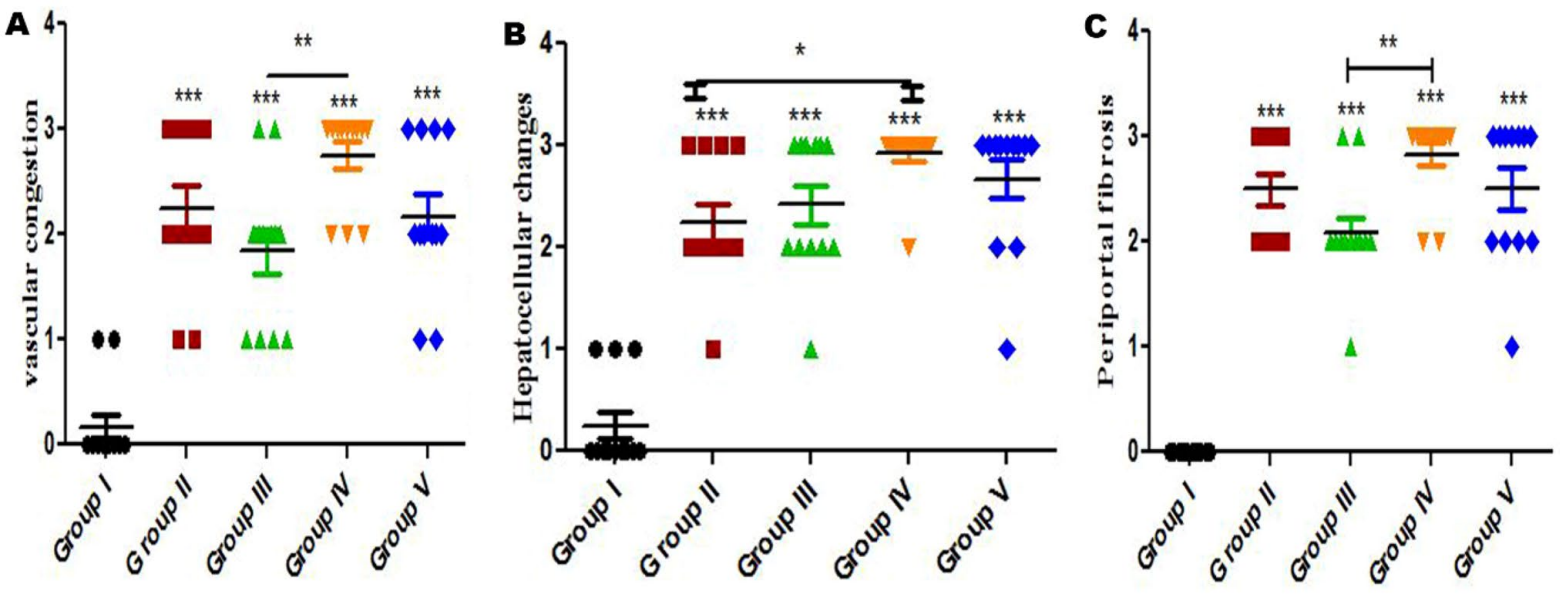
Histomorphometry graph showing the semiquantitative measurements of rats liver alterations between the experimental groups. Plots showing (**A**) vascular congestion, (**B**) hepatocellular changes, and (**C**) periportal fibrosis among the experimental groups. Data were represented as mean ± standard error. Significant differences versus the control Group determined through one-way ANOVA with Tukey’s post hoc test are marked by different asterisks: *P ≤ 0.05, **P ≤ 0.01, ***P ≤ 0.001).

Based on histopathology scoring, the severity of vascular congestion (Fig. 13A), hepatocellular changes (Fig. 13B) and periportal fibrosis (Fig. 13C) were significantly increased (*P* ≤ 0.05) in all treated experimental groups compared with the control Group.

Probiotics may theoretically be implicated in inducing different side effects as:

Systemic infections, excessive immune stimulation in susceptible individuals, gene transfer and deleterious metabolic activities; those in according to a 2002 report jointly released by the World Health Organization (WHO) and the Food and Agriculture Organization (FAO) of the United Nations (http://www.fda.gov/ohrms/dockets/dockets/95s0316/95s-0316-rpt0282-tab-03-ref-19-joint-faowhovol219.pdf).

Our experimental results showing several systemic disturbances in liver and kidney cellular metabolism which alter its morphology in experimental groups, in addition to several vascular and inflammatory changes. Our findings were in agreement with previously recorded studies, which found many side effects after probiotic administration^41–62^.

Lactobacillus bacteremia and overt sepsis have previously been observed in association with S. boulardii [cerevisiae], Lactobacillus GG, Bacillus subtilis, Bifidobacterium breve, or combination probiotics^41–47^. Endocarditis has also been observed as a result of both Lactobacillus and Streptococcus probiotics^48,49^. Lactobacillus rhamnosus has also been linked to the formation of an abscess on two occasions^50,51^.

Because probiotics have been found to alter both the innate and adaptive immune systems, including cytokine release and dendritic cell function^52,55^, there has been some worry regarding the risk of overstimulating the immune system in some individuals, possibly leading to autoimmune phenomena or inflammation.

It was confirmed that, plasmids carrying genes giving resistance to tetracycline, erythromycin, chloramphenicol or lincosamide, macrolide, streptomycin, and streptogrammin are found in lactic acid bacteria^56,58^. Transfer of conjugation from enterococci to lactobacilli and lactococci can occur in animals' guts as well as in vitro; however, transfer to lactobacilli is uncommon ^59^.

One clinical trial raised serious concerns about the safety of probiotics^60^. The subjects in the probiotic arm of the trial had a greater mortality rate, which was ascribed to intestinal ischemia. The scientists speculated that probiotic bacteria delivery raised the oxygen demand in the gut mucosa, despite the fact that blood flow was already modest. Alternatively, the probiotics could have caused an inflammatory response in the small bowel, resulting in decreased capillary blood flow. In patients with pancreatitis, two previous smaller-scale investigations (later combined to boost power)^61,62^ showed a reduction in septic sequelae, surgical intervention, and infected necrosis in patients with pancreatitis given a symbiotic containing lactic acid bacteria and fiber.

As a result, many investigators have engaged in discussions with regulators concerning the challenges these policies pose to those seeking to advance scientific knowledge regarding the efficacy and safety of these products. It is critical to validate the identity of the organism through molecular testing at a reference laboratory if an infection appears to be caused by a probiotic strain and this aspect will be taken in our consideration in the following research work.

## Conclusions

White soft cheese is the most consumed dairy product in Egypt and North Africa. Adding low levels of salt when making this type of cheese may threaten the safety of the cheese during storage, so many manufacturers resort to storing cheese under refrigeration. In this study, some strains of lactic acid bacteria were used to improve the chemical composition and physical properties of the cheese as well as its capability to produce lactic acid that may help in enhancing the preserving ability of the cheese. Where, the cheese treatments fulfilled the purpose of adding lactic acid bacteria, as *L. rhamnosus* cheese had the highest values of titratable acidity. Multiple histopathological alterations were present in all experimental groups aside from the control rats. The rat kidneys of the experimental groups showed renal vascular congestion particularly in the cortical region. The glomerular changes consist of atrophy, distortion, hypocellularity of the glomerular tuft, and focal lymphoid cell reactions. The renal tubular epithelium showed a series of degenerative changes up to necrosis. The hepatic tissues exhibited variable hepatic injury as a thickening in the Glisson capsule, dissociation, and disorganization of hepatic cords. Hepatocellular vacuolar degeneration, presence of focal areas of nodular hyperplasia, the hyperplastic cells mixed with lymphocytic infiltration, congestion in the portal vein, periportal fibrosis, and edema with the presence of newly formed nonfunctional bile ductulus.

On the basis of histopathology scores, the severity of renal and hepatic changes was significantly increased in all treated experimental groups compared with the control Group.
