# Author Correction: Effect of cultured white soft cheese on the histopathological changes in the kidneys and liver of albino rats

**DOI:** 10.1038/s41598-022-08515-3

**Published:** 2022-03-14

**Authors:** Khaled H. Salman, Fatma Abo Zakaib Ali, Ruwaida Elhanbaly

**Affiliations:** 1 Department of Dairy Science, Faculty of Agriculture, AlAzhar University, Assiut, 71524 Egypt; 2 Department of Pathology and Clinical Pathology, Faculty of Veterinary Medicine, Sohag University, Sohâg, 82524 Egypt; 3 Department of Anatomy and Histology, Faculty of Veterinary Medicine, Assiut University, Assiut, 71515 Egypt

Correction to: *Scientific Reports* 10.1038/s41598-022-06522-y, published online 15 February 2022

The original version of this Article contained errors in the legends of Figures 10, 11 and 12.

In the legend of Figure 10,

“Photomicrograph of the rat kidneys of Group III (treated with basal diet 70% + cheese containing *L. helveticus* 30%).”

now reads:

“Photomicrograph of the rat livers of Group III (treated with basal diet 70% + cheese containing *L. helveticus* 30%).”

In the legend of Figure 11,

“Photomicrograph of the rat kidneys of Group IV (basal diet 70% + cheese containing *L. rhamnosus* 30%).”

now reads:

“Photomicrograph of the rat livers of Group IV (basal diet 70% + cheese containing *L. rhamnosus* 30%).”

In the legend of Figure 12,

“Photomicrograph of the rat kidneys of Group V (basal diet 70% + cheese containing *S. thermophilus* S_3855_ 30%).”

now reads:

“Photomicrograph of the rat livers of Group V (basal diet 70% + cheese containing *S. thermophilus* S_3855_ 30%).”

The original Article has been corrected.

